# Halophytes of the Mediterranean Basin—Underutilized Species with the Potential to Be Nutritious Crops in the Scenario of the Climate Change

**DOI:** 10.3390/foods10010119

**Published:** 2021-01-08

**Authors:** Agatha Agudelo, Micaela Carvajal, María del Carmen Martinez-Ballesta

**Affiliations:** 1 I+D Department, Sakata Seed Iberica, Pl. Poeta Vicente Gaos, 46021 Valencia, Spain; agatha.agudelo@sakata.eu; 2 Biotechnology Department, Universidad Politécnica de Valencia, Camino de Vera s/n, 46022 Valencia, Spain; 3 Group of Aquaporins, Centro de Edafología y Biología Aplicada del Segura-Consejo Superior de Investigaciones Científicas, P.O. Box 164, Espinardo, 30100 Murcia, Spain; mcarvaja@cebas.csic.es; 4 Department of Agronomy Engineering, Universidad Politécnica de Cartagena, Paseo Alfonso XIII, 48, 30203 Cartagena, Spain

**Keywords:** *Atriplex halimus*, *Cakile maritima*, minerals, phenolic compounds, *Salicornia fruticosa*, salinity

## Abstract

Halophyte plants are adapted to saline environments and represent a novel type of crops given their possible uses at both culinary and industrial levels. In this work, the nutritional quality of different Mediterranean halophyte species, *Atriplex halimus*, *Salicornia fruticosa*, and *Cakile maritima*, was evaluated under conditions of high salinity. For this, plants were grown at different NaCl concentrations (0, 100, 200, and 300 mM) and the contents of proteins, total lipids, polyphenols, and mineral elements were analyzed as well as growth. Of the three species, *C. maritima* was the most sensitive to salt stress and therefore showed the highest phenolic compounds content. By contrast, whereas salinity increased the amounts of proteins and phenolics with respect to the control in *A. halimus* and *S. fruticosa*, it decreased them in *C. maritima*. Plants of *A. halimus* accumulated higher amounts of Na^+^ in their leaves, but the level of this ion, considering human consumption, was below that of other culinary halophyte species. In conclusion, all the results indicate that these three halophyte species grown at high salt levels represent optimal crops for—new foodstuff—production as green salt or spice due to their nutritional potential.

## 1. Introduction

Soil and water salinization is an increasing problem worldwide [1,2]. Salinization has been widely studied and previous authors have already alerted society of how this problem can influence the future of humanity. While global food production will need to increase by 38% by 2025 and by 57% by 2050, to satisfy the growing population, about 15% of the total land area of the world is estimated to have been degraded by soil erosion and physical and chemical degradation, including soil salinization [3]. Therefore, progressive salinization of arable land has become one of the most important and global factors contributing to land degradation, especially in arid and semi-arid zones.

Salinity affects plant growth and water and nutrient uptake of glycophytes [4]. The adverse effects of salinity on plant growth are (1) water stress produced by the decrease in the osmotic potential of the soil solution, (2) nutritional imbalance, (3) salt stress caused by the specific effect of ions, or (4) the combination of any of these factors [5,6]. However, halophytes can grow at high levels of salinity, being defined as plants that live in naturally saline habitats or that complete their life-cycle at a salt concentration of at least 200 mM NaCl [7]. Facing the increasing salinization throughout the world, domestication of these plants has been suggested as a possible solution, using them as potential crops in “saline agriculture” or in new foodstuff [4,8,9,10]. There are two factors that make halophytes of special interest to be considered in the food industry: First, their economic potential, since their productivity in high-salinity and low-water intake environments, is much higher than that of ºtraditional edible species. Second, their nutritional value in terms of their protein, phenolic, lipid contents and the great quantity of minerals, such as potassium, calcium, and magnesium, and other bioactive compounds, [11,12,13].

Salinity is an environmental stress that has been identified as a possible trigger for oxidative reactions in plants. The reactive oxygen species (ROS) generated in these reactions react with biological molecules causing cellular damage, metabolic disorders, and senescence processes. In order to decrease the ROS content, halophytes have been reported to increase the synthesis of antioxidant molecules such as phenolics compounds [11,14,15].

Phenolic compounds are secondary metabolites that are derivatives of the pentose phosphate, shikimate, and phenylpropanoid pathways in plants [16]. These compounds have been widely studied due to their preventive and therapeutic properties including anti-inflammatory, anti-allergenic, antioxidant, anti-atherogenic, anti-microbial, anti-thrombotic, and cardioprotective properties [15]. These beneficial properties contribute to the rising interest in the use of halophytic species as functional food [13,17,18]. In fact, the use of phenolic compounds as a supplement has already been studied in several foods and food models systems [19].

Among the halophytes, *Cakile maritima* (sea rocket) is a plant that tends to develop succulence under salt stress. This plant has been used as a flavoring agent for salads and as a dried ingredient in flours to make bread [20]. Moreover, it has been exploited for the appreciable amounts of oil in its seeds (40% on dry weight basis) [21]. Moreover, during the past decade several studies have been carried out in Salicornia (*Salicornia fruticosa*) in order to trial its inclusion in several foods. Salicornia-derived salt and its production has been described already [22] and also, as a functional ingredient, it has been added to reduced-salt cooked sausages [23], and sport beverages [24] among other products. As a vegetable, young Salicornia shoots have been introduced into the European gourmet market [25]. Finally, *Atriplex halimus* leaves have traditionally been used as a condiment due to its salty flavor [26]. However, their consumption has mainly occurred at times when other sources of food were unavailable [27]. This might be the reason why very few examples can be found regarding the use of *A. halimus* as human food.

Therefore, it is of great interest to evaluate the bioactive compounds and minerals in these halophytes (*C. maritima*, *S. fruticosa* and *A. halimus*) taking into consideration the influence of different levels of salinity on their yield, in terms of biomass. This is the aim of this work. Precisely, the response of these three halophytes to increasing levels of salinity in the nutrient solution at an early stage of growth was evaluated and the nutritional analysis of minerals, phenolic compounds, proteins, and lipids was performed.

## 2. Material and Methods

One-month-old plants of three halophyte species—*Atriplex halimus*, *Cakile maritima*, and *Salicornia fruticosa*—were provided by Viveros Muzalén (Murcia, Spain) The plants were transferred to a controlled-environmental chamber, with a 16-h light and 8-h dark cycle with temperatures of 25 and 20 °C and relative humidities of 60% and 80%, respectively. Photosynthetically active radiation (PAR) of 400 μmol m^−2^ s^−1^ was provided by a combination of fluorescent tubes (Philips TLD 36 W/83, Jena, Germany, and Sylvania F36 W/GRO, Manchester, NH, USA) and metal halide lamps (Osram HQI, T 400 W, Berlin, Germany). The plants were placed in 15-L containers with continuously-aerated Hoagland nutrient solution [28]. After 1 month of growth, different saline treatments (0, 100, 200, and 300 mM NaCl) were applied for 2 weeks.

### 2.1. Relative Growth Rate

To calculate the RGR, the formula described by Hunt et al. [29] was used.

$$\mathrm{RGR}=\frac{\ln\left(W2\right)\;-\;\ln\left(W1\right)}{t2\;-\;t1}$$

where W1 and W2 are the dry weight or ash-free dry weight of the plants at the initial and final harvests, respectively, and t2 − t1 is the time in days between the two harvests.

### 2.2. Analysis of Mineral Elements

The concentrations of macronutrients and micronutrients were analyzed in oven-dried samples of plant material (young leaf and stem tissues) which had been ground finely in a mill grinder (IKA model A10, Staufen, Germany) to give particle sizes of 0.5 to 0.7 mm. The samples were digested in a microwave oven (CEM Mars Xpress, Mattheus, NC, USA) by HNO_3_–HClO_4_ (2:1) acid digestion. The elemental analysis was carried out using a Perkin–Elmer (Waltham, MA, USA) 5500 model ICP emission spectrophotometer (Iris Intrepid II, Thermo Electron Corporation, Franklin, TN, USA), at 589 nm. The concentrations were expressed as mg kg^−1^ DW.

### 2.3. Phenolic Compounds

Freeze-dried powder (50 mg) from leaf and stem tissues was extracted in 1.5 mL of 70% MeOH for 30 min at 70 °C, vortexing every 5 min to improve extraction. Afterwards, the extract was centrifuged (20 min, 10,000× *g*, 4 °C) (Sigma 1–13, B. Braun Biotech Intl., Osterode, Germany). The supernatants were collected and the methanol was removed using a rotary evaporator; the dried residue was reconstituted in ultrapure water to 1 mL and filtered through a 0.2-µm inorganic membrane filter (ANOTOP10 plus, Whatman, Maidstone, UK). Each sample (20 µL) was analyzed in a Waters HPLC system (Waters Cromatografía S.A., Barcelona, Spain), consisting of a W600E multisolvent delivery system, inline degasser, W717plus autosampler and W2996 PAD. The compounds were separated in a Luna C_18_ column (25 × 0.46 cm^2^, 5 μm particle size; Phenomenex, Macclesfield, UK) with a security guard C_18_-ODS (4 × 30 mm^2^) cartridge system (Phenomenex). The mobile phase was a mixture of water/trifluoroacetic acid (99.9:0.1, *v*/*v*) (A) and acetonitrile/trifluoroacetic acid (99.9:0.1, *v*/*v*) (B). The flow rate was 1 mL min^−1^ in a linear gradient, starting with 1% B for 5 min to reach 17% B at 15 min, which was maintained for 2 min, then 25% B at 22 min, 35% B at 30 min, 50% B at 35 min and 99% B at 40 min. The monitored compounds eluted off the column in 35 min. The chromatograms of the phenolic compounds were recorded at 330 nm. The abundance of each tentatively identified polyphenol was calculated by measuring the area of the each peak and quantified using external standards: caffeoylquinic acid derivates using chlorogenic acid (Sigma-Aldrich, St. Louis, MO, USA), flavonoids with quercetin-3-rutinoside (Sigma-Aldrich) and sinapic acid derivatives using sinapinic acid (Sigma-Aldrich). The contents of phenolic compounds were expressed as mg g^−1^ DW (dry weight).

### 2.4. Protein and Total Lipid Analysis

The method of Bradford was used to determine protein content [30], using the Bio-Rad reagent with BSA as standard, and was expressed as mg g DW^−1^. Total lipids were extracted according to the method of Folch et al. [31], including some modifications. In order to denature phospholipases 100 mg of fresh material (leaves and stems) were soaked in boiling water for 5 min and then homogenized in a chloroform–methanol mixture (2:1, *v*/*v*). The homogenate was centrifuged at 3000 rpm for 15 min. The lower (chloroformic) phase, containing lipids, was isolated and evaporated under N_2_ gas. The residue was weighed for determination of total lipid fraction.

### 2.5. Data Analysis

Statistical analyses were performed using Statgraphics XVII-X64 for Windows. Significant differences among the mean values were determined at *p* ≤ 0.05, according to Tukey’s test.

## 3. Results

### 3.1. Relative Growth Rate

**Figure 1:**
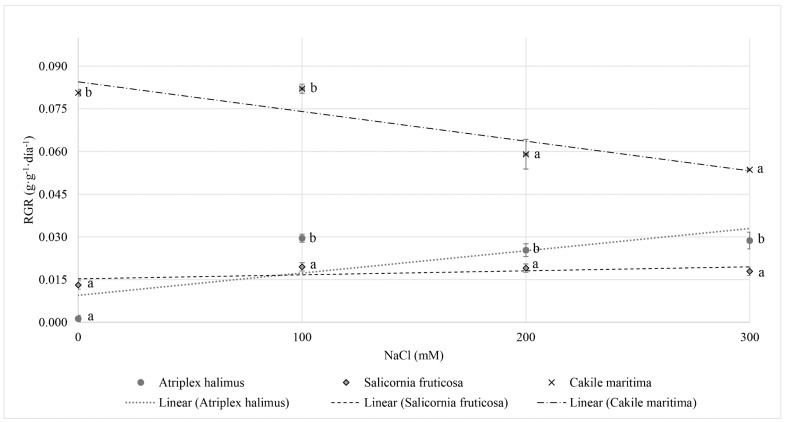
Relative Growth Rate, RGR (g·g^−1^ DW·day^−1^) of *Atriplex halimus*, *Cakile Maritima*, and *Salicornia fruticosa* under different salinity treatments (0, 100, 200, and 300 mM NaCl) after two weeks of treatment. Means (*n* = 5 ± SE) with different letter are significantly different at *p*-value < 0.05, different lower-case letters are significantly different among the same species under different treatments, and different capital letters are significantly different among different species under the same treatment. Tukey HSD test was used for mean comparison.

Biomass (expressed as gr per plant) was evaluated following the two-week exposure to increasing salinity (0–100–200–300 mM NaCl) (Figure 1). *Salicornia fruticosa* showed the greatest RGR for all treatments, followed by, *Atriplex halimus*, and *Cakile maritima*. However, while the RGR in *Atriplex halimus* and *Salicornia fruticosa* was enhanced with salinity, in *Cakile maritima*, RGR was decreased with the increase in salinity.

### 3.2. Mineral Elements

As expected, *Atriplex halimus* and *Salicornia fruticosa* accumulated the highest amount of Na^+^. The *Cakile maritima* tissues showed the highest values of Ca^2+^, P, and S (Table 1). Moreover, in this species, the Na^+^ content increased with the enhance in salinity and this was accompanied by a decrease in the uptake of Ca and Mg and an imbalance in other elements. This imbalance effect was not found in *Atriplex halimus* or *Salicornia fruticosa*, which maintained the same levels of Ca^2+^, Mg^2+^, P, and S at moderate and high salinity.

Micronutrients were also determined in the studied halophytes (Table 2). The Fe content increased with salinity in *Atriplex halimus* and *Salicornia fruticosa* at 300 mM and 100 mM NaCl, respectively, but remained unaltered in *Cakile maritima*. Plants of *Cakile maritima* showed the highest Zn levels, being 10-fold higher than in *Atriplex halimus*; however, salinity did not modify Zn content in all species. Only in *Salicornia fruticosa*, Mo, B, and Mn contents increased by salinity; in the other species they were maintained or reduced, depending on the salt concentration.

### 3.3. Phenolic Content

In *Atriplex halimus*, a total of 12 phenolic compounds corresponding to 5 phenolic acids derivatives and 7 flavonoids were detected. While in *Cakile maritima*, a total of seven peaks corresponding to five phenolic acids derivatives and two flavonoids were detected in *Salicornia fruticose* a total of four phenolic compounds corresponding to one phenolic acid derivatives and three flavonoids were found. Total phenolic compounds were calculated as the sum of all of them.

*Cakile maritima* showed the highest level of phenolic content, but there was a negative correlation with the NaCl concentration, with the exception of flavonoids, whose content did not vary among the treatments (Table 3). In *Atriplex halimus*, there was a reduction in sinapic acid derivatives and an increase in flavonoids and total phenolics with the increase in the NaCl concentration. Even though *Salicornia fruticosa* had a greater content in chlorogenic acid derivatives and flavonoids than *Atriplex halimus*, it had the lowest levels of total phenolics. No sinapic acid derivatives were found and the greatest levels of phenolics were detected at 100 mM NaCl.

### 3.4. Protein and Lipid Content

Total protein content was evaluated among all treatments and species (Table 4). The highest total protein content was identified for *Atriplex halimus* at moderate salinity (100 mM NaCl), followed by *Salicornia fruticosa* and *Cakile maritima* presented the lowest content, under all salinity treatments. No significant correlation was found between the protein content and salinity, for any of the species studied.

Total lipid content, expressed as mg g^−1^ DW, did not show significant differences among the treatments in each specie. It was highest in *Cakile maritima* plants while *Atriplex halimus* and *Salicornia fruticosa* showed similar lipids contents.

## 4. Discussion

In most plants, growth gradually decreases as salinity increases above the threshold of salinity tolerance [32]. The exceptions are halophyte plants, whose growth can be stimulated by low or moderate salinity, but the threshold salt concentration that stimulates or limits growth strongly depends on the genotype [33]. Thus, despite the fact that Debez et al. [34] found a slight RGR enhancement at moderate salinity in *Cakile maritima*, no significant differences in RGR were detected in our *Cakile maritima* plants at 100 mM NaCl, relative to the control. This slight non-alignment may be due to the different ecotype used in their experiments. In fact, in other reports a correlation between the *Cakile maritima* ecotype and the response to salinity regarding growth was found [18,35,36,37]. However, in our work, at high levels of salinity the RGR of *Cakile maritima* was reduced with respect to the control (by ca. 27% at 200 mM NaCl and by 34% at 300 mM NaCl). These results are consistent with previous findings [34,36,38,39] indicating the facultative character of this halophyte, since its growth is not stimulated by salinity, but it can tolerate moderate salt stress.

On the other hand, *Atriplex halimus* showed a halophytic character with almost no growth in the non-salt treatment and a significant improvement in plant performance under NaCl treatments. These results correlate well with previous studies of *Atriplex* halimus under different NaCl concentrations, which showed that *Atriplex halimus* growth was not negatively affected by salinity until the NaCl concentration reached 400 mM [40,41,42]. In fact, according to Belkheri and Mulas [43], there is a threshold at 300 mM NaCl above which leaf RGR decreases while stems and roots continue growing until the NaCl concentration reaches 600–800 mM.

Our results for *Salicornia fruticosa*, are in consonance with previous results showing an enhancement of its growth under salinity compared with control conditions [17]. However, we found RGR values lower than those reported in the literature [44,45,46]. These differences can be explained in part by the differences in the genotype used and in the experimental conditions, especially the duration of the salinity treatments and the growth chamber conditions. It is worthwhile noting that Katschnig, Broekman, and Rozema [44] reviewed 20 papers that study *Salicornia* spp. growth and for the majority of them (16 out of 20) an enhancement in performance was found at around 100 mM NaCl, considering this concentration optimal for *Salicornia* growth. Taking into account that for most of the ca. 5000 crop species that are cultivated throughout the world, growth and yield are severely affected at a soil salt level below 0.1% (17 mM NaCl) [47], the evidence from our study supports the idea of exploring halophytes for economic vegetable production, as has been suggested previously [48]. All the ecotypes studied here can be grown in saline areas without reductions in their biomass yield.

One of the main concerns regarding the use of halophytes as new food crops is the fact that they accumulate high amounts of Na^+^ and Cl^−^ in their edible parts. *Atriplex halimus* copes with salinity mainly by excreting Na^+^ and Cl^−^ into vesiculated hairs, which deposit them in crystals that form on the leaves [27,43]. However, *Salicornia fruticosa* and *Cakile maritima* prevent the excessive accumulation of Na^+^ in the cytosol by using compartmentation in the vacuole; therefore, the Na^+^ remained in the tissue.

The World Health Organization (WHO) recommends that the daily intake of Na^+^ does not exceed 2000 mg day, since it can cause pathologies such as hypertension and cardiovascular diseases [49]. Similarly, the nutritional guidelines in and outside Europe reflect the importance to strongly decrease the Na^+^ intake [50], considering a maximum consumption of 5 g per day in most countries including Spain [51].

Based on our experiments, considering an average moisture percentage of 85% for *Atriplex halimus* and *Salicornia fruticosa* and 92% for *Cakile maritima* (data not shown), the consumption of 100 gr of these fresh vegetables would suppose a maximum intake of 1105.35 mg of Na^+^ (for *Atriplex halimus* plants treated with 300 mM NaCl). This amount is below or similar to those obtained for other halophyte species of culinary interest, such as *Arthrocnemum macrostachyum*, *Sarcorconia perennis alpini* and *Salicornia ramosissima*: 2049, 1029, and 1393 mg of Na^+^, respectively [52]. Other foods commonly used as a source of Na^+^, such as seaweed, have been found to accumulate up to 3960 Na^+^ mg 100 g^−1^ DW [53]. In this sense, it is worth highlighting that for *Salicornia fruticosa* and *Cakile maritima* at 100 mM NaCl—when the accumulation of Na was lower, 2733.93 and 2833.61 mg 100 g^−1^ DW, respectively—an increase in growth occurred. However, considering WHO and European recommendations concerning Na daily intakes, the use of these species as fresh vegetables is not recommended, but they can be used as dish accompaniment or as new condiments. Thus, a high nutritional “green salt” based in two halophytes extracts was previously obtained (Antunes et al., 2018). Moreover, a freeze-dried powder of sea fennel has been elaborated as a flavoring spice (Renna and Gonnella, 2012). Similar uses can be proposed by our halophyte plants, but new agronomical and post-harvest strategies must be developed in order to reduce salinity levels in the plant edible parts, as indicated Renna and Gonnella (2018). Furthermore, the use of young leaves can ameliorate Na^+^ accumulation regarding the older ones [54,55,56].

However, we should interpret carefully the results for the Na^+^ concentration in the leaves of the plants since the time of NaCl exposure is the main determinant of the Na^+^ concentration in tissues. Thus, the short exposure time could explain our lower levels of Na^+^ in *Salicornia fruticosa* compared with those reported by Lv et al. [57]. In contrast, our findings for *Salicornia fruticosa* correlate well with those of Ushakiva et al. [58] and Tikhomirova et al. [59] for similar periods of exposure to salinity. Therefore, a controlled NaCl exposition during halophyte crop production cannot be ruled out.

At high salt concentrations, the competition between Na^+^ and other ions increases and a nutrient imbalance in the plant could occur. Consequently, although halophytes accumulate Na^+^, the K^+^, Ca^2+^, and Mg^2+^ contents may decrease. In *Atriplex halimus*, most authors have noted a decrease in the leaf K^+^, Mg^2+^, and Ca contents, while the Na content increases, with increasing salinity [42,60]. However, in our plants, although the concentrations of the rest of the macronutrients were reduced with by salinity, relative to control, the K^+^, Ca^2+^, P and S concentrations were maintained in all the salinity treatments, indicating that the increment in Na ions did not impede the uptake or translocation of other cations. This behavior was previously found in *Salicornia fruticosa* and *Cakile maritima*, with an effective balance for the other macronutrients being maintained in spite of the rise in the external NaCl concentration [17,57].

From a nutritional point of view, it is important that the Na^+^/K^+^ ratio remains low, since diets with a high Na^+^/K^+^ ratio have been related to various cardiovascular diseases [61]. High concentrations of K^+^ were found in *Atriplex halimus* and *Salicornia fruticosa* (ranging from 1139.77 to 2158.81 mg 100 g^−1^ DW) under saline stress conditions, exceeding the values reported for other halophytes of culinary interest, which ranged from 892 to 1580 mg 100 g^−1^ DW [61]. Moreover, the concentration of macronutrients as Ca^2+^, Mg^2+^, P, and S (essential elements for humans) must be considered in vegetables, since these minerals are required at doses higher than 50 mg/day in the adults (the amounts recommended by the Food by the Food and Nutrition Board of the Institute of Medicine in Washington DC, 2002 [62]). The Ca^+2^ levels of *Cakile maritima* were higher than for the rest of the halophytes studied, comparable to others of interest (52–62 mg 100 g^−1^ DW [17], and within the range of other horticultural plants: between 2 and 190 mg 100 g^−1^ FW) [63].

Zinc and iron deficiencies are the most common and widespread nutritional deficiencies in the world [64]. *Cakile maritima* plants in our study presented levels of Zn ranged from 2.36 to 2.96 mg per 100 gr DW. Zn content of lettuce leaves were ranged from 3.41 to 4.68 mg/100 g DW depending on the variety and location [65,66]. Other baby leaf vegetables contained 0.84 mg/100 gr FW of Zn [67]. Thus, our *Cakile maritima* plants are a good source of Zn and the three species tested here, when cultivated in a hydroponic system, did not accumulate amounts of metals above those recommended by the Food and Drug Administration (FDA).

Previous findings suggested a higher phenolic content in *Cakile maritima* plants obtained from extreme climatic conditions in terms of salinity, low rainfall, and high radiation [11]. However, we found a negative correlation between increasing salinity and the total phenolics content, in consonance with analysis carried out under controlled growth conditions [18]. This variation in the response to salinity stress has been related to the ecotype used and the bioclimatic circumstances [37]. Taking into consideration the reduction in phenolics accumulation and the substantial reduction in growth caused by salinity, it seems that *Cakile maritima* is not the best candidate as a source of biochemicals components under salt stress. However, the levels of phenolics are elevated regarding other halophytes in spite of salinity reduction. Soxhlet extracts of *Chrtymum maritimum* leaves [68], another promising food halophytic plant, showed a total phenolic compound levels ranged from 3.68 to 4.33 mg GAE g^−1^ DW and a content of total flavonoids from 1.70 to 1.87 (mg of CE g^−1^ DW), which values were similar to our *Cakile maritima* plants.

Flavonoids, in addition to the antioxidant properties of phenolic compounds, have other several potential health-promoting activities, including anti-allergic, anti-inflammatory, anti-microbial, and anti-cancer properties [69]. Thus, the use of edible parts of *Cakile maritima* must be considered as a source of these antioxidant compounds in the search of new salt tolerant crops with moderate–high nutritional value and agri-food industrial applications. Conversely, our results with *Atriplex halimus* show an increase in flavonoids and total phenolics with the increment in the salt concentration, while RGR increased at the same time. This is in agreement with Bendaly et al. [42] and Boestfleisch et al. [70], who reported that these correlations continue up to 400 mM NaCl, from which point growth and antioxidants were affected is reduced. Nevertheless, there is considerable literature on how salinity stress seems to promote the accumulation of phenolic compounds in *Ariplex halimus* [12,42,70]. For some *Salicornia species*, an increase in the phenolics content with increasing salinity has been reported [70]. However, our results show a peak around 100 mM NaCl. Even though the total phenolics content decreased when the salinity passed this point, at 300 mM NaCl the flavonoids content was still higher than in control plants, in contrast to the results of Mishra et al. [13]. These authors also found an increment in flavonoids with enhanced salinity.

*Atriplex halimus* had the highest amounts of proteins under salinity, followed by *Salicornia fruticose*, their protein contents being higher than that obtained for the Nori alga grown at 100 mM NaCl (20 g/100 g DW); this alga is considered as a new foodstuff and is the most consumed seaweed in the world. Significantly, *Atriplex* spp. are known to have a high protein content, around 14–21% [71,72,73] which is higher than well-established crop species such as alfalfa. That is why these species have been studied to be as a partial replacement for other traditional forage sources which are not as well adapted as *Atriplex* spp. to cultivation in arid and semiarid areas [9,74]. Remarkably, our results showed up to a 32% crude protein content, which may be related to the early plant stage in which the analysis was carried out. In *Salicornia fruticosa*, the protein content was also in agreement with previous studies with different *Salicornia* spp. [14,17,75]. In fact, *Atriplex halimus* and *Salicornia fruticosa* showed almost twice total protein content of *Cakile maritima* and the consumption of *Atriplex* spp. have been reported in times of scarcity of other vegetable foods as a good source of protein [27]. An analysis of the fatty acids in these three halophytes was previously reported [76]. In general, halophytes have been reported to provide nutritional value due to their lipid composition [77], pointing that lipids were increased when they were grown in farms when compared to conspecifics from the wild conditions. Furthermore, previously *Cakile maritima* was described as a species with a high degree of unsaturated fatty acids when compared glycophytes plants [78]. The results showed that the most abundant fatty acids for the three species were linoleic, α-linoleic, and arachidic acids, which are part of the omega-3 and omega-6 series, the most important in human nutrition. The results obtained in other works point to the lipid composition in this plants attempt to be healthy. Thus, the elevated lipid content in Cakile plants, even at high salinity, represents an added value to its nutritional profile, since main lipids have known bioactive properties. Accordingly, the specific composition of the lipids should be further determined.

## 5. Conclusions

Taking into account that from the about 5000 crops that are cultivated throughout the world, in most of them, growth and yield are severely affected under 0.1% soil salinity [47], the evidence from this study points out towards the idea of exploring halophytes for economic vegetable production, as it has been suggested in previous literature [48]. *Atriplex halimus* and *Salicornia fruticosa* were proved to have the best growth and biomass yield under salinity stress and so should be studied further for their application in degraded soils and coastal areas affected by salinity.

All the above results contribute to the promotion of halophytes crops as a source of valuable material for foodstuff production. Thus, these plant species, that due to their characteristics can tolerate saline soils and saline irrigation waters, may be an alternative to conventional crops for foodstuff, given their mineral nutritional potential. As some of them may result hyperaccumulators, heavy metals contamination of soil has to be considered when deciding whether to cultivate them. In any case, these species did not accumulated amounts of metals over than those recommended by Food and Drug Administration (FDA), when they are cultivated in a hydroponic system under controlled conditions.

*Atriplex halimus* may provide an acceptable level of proteins that can fulfil the growing demand to reduce the animal protein consumption. While *Cakile maritima* plants resulted less tolerant to salinity, they constitute a source of antioxidant compounds as total phenolics, especially flavonoids, with important contributions to human health. Thus, the use of these species as herbal salts or salt extracts for foodstuff substituting NaCl cannot be ruled out. Moreover, there are few directresses on how efficiently they must be cultivated, and research must be conducted through this direction. When taken into consideration the challenges future generation will face in terms of soil degradation, climate change, and increasing population, it is more probable that these species will be taken into consideration as new cash crop halophyte.

## Figures and Tables

**Table 1:** Macronutrient content in the leaves and stems of *Atriplex halimus*, *Cakile Maritima*, and *Salicornia fruticosa* grown with different salinity doses (0, 100, 200, and 300 mM NaCl) for two weeks. Cation concentrations are expressed as mg/100 g^−1^ DW (mean ± SE, *n* = 5). Values followed by different letters are significantly different at *p*-value < 0.05; upper-case letters indicate the differences among the salinity treatments for the same species, lower-case letters indicate the differences among the species for the same treatment. The Tukey HSD test was used for the comparison of means.

Species	Treatments (Mm)	Na	K	Ca	P	Mg	S
*Atriplex halimus*	0	989.37 ± 192.08 Ba	5725.03 ± 953.91 Bb	565.42 ± 83.93 ABb	140.19 ± 19.00 Ab	224.45 ± 36.20 Bb	127.50 ± 19.87 Ab
	100	4472.24 ± 665.96 Ab	2158.81 ± 251.95 Ba	93.60 ± 12.28 Aa	73.78 ± 9.29 Aa	70.11 ± 8.48 Aa	57.39 ± 7.33 Aa
	200	6507.30 ± 119.09 Bc	2029.97 ± 585.11 Aa	79.19 ± 13.19 Aa	85.92 ± 10.62 Aa	70.94 ± 17.06 Aa	80.18 ± 9.49 Aab
	300	7369.31 ± 162.34 Cc	1821.36 ± 44.51 ABa	133.83 ± 1.37 Aa	123.29 ± 1.65 Bab	174.46 ± 3.46 Ab	94.31 ± 1.69 Aab
*Cakile maritima*	0	23.71 ± 0.29 Aa	2940.52 ± 14.48 Ad	787.74 ± 5.91 Bc	209.10 ± 0.51 Ba	116.89 ± 0.99 Ac	243.37 ± 0.59 Bd
	100	2833.61 ± 16.27 Ab	1047.22 ± 20.90 Aa	370.31 ± 6.61 Bb	291.72 ± 8.44 Bc	69.33 ± 1.50 Ab	179.71 ± 4.03 Bc
	200	3422.74 ± 84.77 Ac	1295.66 ± 34.23 Ab	305.52 ± 7.95 Ba	236.29 ± 3.85 Bb	57.26 ± 1.60 Aa	154.84 ± 1.97 Bb
	300	3528.19 ± 49.49 Ac	1469.93 ± 11.92 Ac	320.34 ± 4.75 Ba	216.63 ± 4.09 Cab	55.79 ± 0.67 Aa	141.32 ± 1.72 Ba
*Salicornia fruticosa*	0	967.90 ± 88.80 Ba	4592.69 ± 460.27 ABb	341.42 ± 33.38 Ab	96.38 ± 9.79 Aa	177.71 ± 17.97 ABa	79.53 ± 7.33 Aa
	100	2733.93 ± 204.68 Ab	1139.77 ± 85.95 Aa	68.41 ± 22.53 Aa	120.11 ± 29.25 Aa	120.94 ± 12.56 Ba	59.35 ± 2.97 Aa
	200	3577.05 ± 146.44 Ac	1350.47 ± 238.35 Aa	57.15 ± 4.20 Aa	114.61 ± 31.05 Aa	98.13 ± 18.88 Aa	54.69 ± 6.13 Aa
	300	4314.28 ± 242.84 Bc	2029.20 ± 198.43 Ba	142.94 ± 37.82 Aa	87.87 ± 11.32 Aa	179.63 ± 55.45 Aa	86.61 ± 12.10 Aa

**Table 2:** Micronutrients content in the leaves and stems of *Atriplex halimus*, *Cakile Maritima*, and *Salicornia fruticosa* grown with different salinity doses (0, 100, 200, and 300 mM NaCl) for two weeks. Cation concentrations are expressed as mg 100 g^−1^ DW (mean ± SE, *n* = 5). Values followed by different letters are significantly different at *p* < 0.05; upper-case letters indicate the differences among the salinity treatments for the same species, lower-case letters indicate the differences among the species for the same treatment. The Tukey HSD test was used for the comparison of means.

Species	Treatments (Mm)	Fe	Zn	Mo	B	Mn
*Atriplex halimus*	0	0.41 ± 0.07 Aa	0.41 ± 0.05 Aa	0.03 ± 0.00 Ab	2.50 ± 0.40 Ab	0.65 ± 0.10 Ab
	100	0.36 ± 0.05 Aa	0.23 ± 0.03 Aa	0.02 ± 0.00 Aa	1.52 ± 0.20 Aab	0.25 ± 0.03 Aa
	200	0.41 ± 0.05 Aa	0.28 ± 0.07 Aa	0.02 ± 0.00 Aab	1.35 ± 0.08 Aab	0.30 ± 0.07 Aa
	300	0.63 ± 0.01 Bb	0.30 ± 0.00 Aa	0.02 ± 0.00 Aab	1.49 ± 0.03 Aa	0.64 ± 0.01 Ab
*Cakile maritima*	0	1.14 ± 0.04 Ba	2.36 ± 0.03 Ba	0.07 ± 0.00 Ba	2.66 ± 0.00 Aa	0.68 ± 0.00 Aa
	100	0.69 ± 0.00 Aa	2.17 ± 0.09 Ba	0.05 ± 0.00 Ba	1.80 ± 0.03 Aa	0.61 ± 0.01 ABa
	200	0.63 ± 0.00 Aa	2.31 ± 0.03 Ba	0.05 ± 0.00 Aa	1.67 ± 0.02 Aa	0.56 ± 0.01 Aa
	300	0.57 ± 0.01 ABa	2.96 ± 0.58 Ba	0.05 ± 0.00 Ba	1.67 ± 0.00 Aa	0.56 ± 0.01 Aa
*Salicornia fruticosa*	0	0.47 ± 0.05 Ac	0.36 ± 0.03 Aa	0.02 ± 0.00 Ac	1.65 ± 0.16 Ac	0.63 ± 0.06 Ac
	100	0.86 ± 0.27 Ab	1.07 ± 0.43 ABa	0.04 ± 0.01 ABa	2.07 ± 0.50 Ab	1.28 ± 0.40 Bb
	200	0.70 ± 0.29 Aab	0.96 ± 0.40 Aa	0.03 ± 0.01 Ab	1.90 ± 0.58 Aa	1.06 ± 0.45 Aa
	300	0.44 ± 0.06 Aa	0.32 ± 0.03 Aa	0.02 ± 0.00 Aab	1.44 ± 0.26 Aa	0.62 ± 0.16 Aa

**Table 3:** Flavonoid glycosides, Sinapic and Chlorogenic acid derivatives, and Total (as the sum of all) in the leaves and stems of *Atriplex halimus*, *Cakile Maritima*, and *Salicornia fruticosa*, (mg g^−1^ DW) under different treatments (0, 100, 200, and 300 mM NaCl). Means (*n* = 5 ± SE) with different letter are significantly different at *p*-value < 0.05; different lower-case letters are significantly different among the same species under different treatments, and different capital letters are significantly different among different species under the same treatment. Tukey HSD test was used for mean comparison.

Species	Treatment (mM)	Sinapic Acid Derivatives	Flavonoid Glycosides	Chlorogenic Acid Derivatives	Total
*Atriplex halimus*	0	0.41 ± 0.01 cB	0.98 ± 0.02 aA	0.49 ± 0.01 bA	1.88 ± 0.04 bB
	100	0.20 ± 0.06 bA	1.33 ± 0.05 bA	0.18 ± 0.01 aA	1.71 ± 0.01 bA
	200	0.02 ± 0.00 aA	1.10 ± 0.01 aA	0.24 ± 0.00 aA	1.37 ± 0.01 aA
	300	0.17 ± 0.02 abA	1.57 ± 0.05 cA	0.52 ± 0.03 bA	2.27 ± 0.09 cB
*Cakile maritima*	0	3.22 ± 0.17 cC	1.50 ± 0.08 aC	0.45 ± 0.04 cB	5.17 ± 0.26 cC
	100	2.02 ± 0.06 bB	1.77 ± 0.06 aA	0.20 ± 0.01 abA	3.99 ± 0.12 bA
	200	2.08 ± 0.05 bB	1.70 ± 0.04 aB	0.27 ± 0.01 bA	4.05 ± 0.09 bB
*Salicornia fruticosa*	0	0.00 ± 0.00 aA	0.38 ± 0.01 aB	0.11 ± 0.00 aB	0.49 ± 0.01 aA
	100	0.00 ± 0.00 aA	1.55 ± 0.07 cA	0.24 ± 0.01 cA	1.79 ± 0.08 cA
	200	0.00 ± 0.00 aA	0.32 ± 0.01 aAB	0.10 ± 0.00 aA	0.42 ± 0.01 aA
	300	0.00 ± 0.00 aA	0.75 ± 0.02 bB	0.20 ± 0.00 bA	0.96 ± 0.02 bA

**Table 4:** Protein and Lipid content in the leaves and stems of *Atriplex halimus*, *Cakile Maritima*, and *Salicornia fruticosa* (mg g^−1^ DW) under different treatments (0, 100, 200, and 300 mM NaCl). Means (*n* = 5 ± SE) with different letter are significantly different at *p*-value < 0.05, different lower-case letters are significantly different among the same species under different treatments, and different capital letters are significantly different among different species under the same treatment. Tukey HSD test was used for mean comparison.

Species	Treatments (mM)	Protein Content	Lipid Content
*Atriplex halimus*	0	292.7 ± 6.7 aB	10.4 ± 0.4 aA
	100	316.4 ± 0.8 bC	10.0 ± 0.8 aA
	200	281.0 ± 2.8 aB	10.7 ± 2.0 aA
	300	320.4 ± 1.3 bB	11.3 ± 0.4 aA
*Cakile maritima*	0	128.0 ± 21.9 aA	21.0 ± 0.7 aB
	100	143.6 ± 5.5 aA	23.1 ± 6.8 aAB
	200	132.5 ± 25.0 aA	19.2 ± 0.6 aB
	300	168.2 ± 47.4 aA	19.6 ± 0.5 aB
*Salicornia fruticosa*	0	271.2 ± 6.9 bB	9.9 ± 0.6 aA
	100	250.9 ± 4.6 abB	10.1 ± 0.9 aA
	200	231.9 ± 3.1 aB	12.1 ± 1.1 aA
	300	263.5 ± 2.6 bAB	12.2 ± 0.3 aA
